# Strawberry *Fragaria x ananassa* cv. Festival: A Polyphenol-Based Phytochemical Characterization in Fruit and Leaf Extracts

**DOI:** 10.3390/molecules28041865

**Published:** 2023-02-16

**Authors:** Karla Salas-Arias, Andrea Irías-Mata, Andrés Sánchez-Kopper, Ricardo Hernández-Moncada, Bridget Salas-Morgan, Fabián Villalta-Romero, Laura A. Calvo-Castro

**Affiliations:** 1 Doctorado en Ciencias Naturales Para el Desarrollo (DOCINADE), Instituto Tecnológico de Costa Rica, Universidad Nacional, Universidad Estatal a Distancia, Cartago P.O. Box 159-7050, Costa Rica; 2 Centro de Investigación en Biotecnología, Escuela de Biología, Instituto Tecnológico de Costa Rica, Cartago P.O. Box 159-7050, Costa Rica; 3 Centro de Investigación en Granos y Semillas, Escuela de Agronomía, Universidad de Costa Rica, San José P.O. Box 2060, Costa Rica; 4 Centro de Investigación y de Servicios Químicos y Microbiológicos, Escuela de Química, Instituto Tecnológico de Costa Rica, Cartago P.O. Box 159-7050, Costa Rica

**Keywords:** *Fragaria x ananassa* cv. Festival, leaves, polyphenols, strawberry

## Abstract

Berry fruits are an important dietary source of health-promoting antioxidant polyphenols. Interestingly, berry leaves of diverse species, including strawberries, have shown higher bioactive phytochemical content in the leaves than in the fruit. Moreover, the vegetative part of the plants is usually discarded, representing a presumably large source of underutilized bioactive biomass. In this investigation, the polyphenol profiles of tropical highland strawberry (*Fragaria x ananassa* cv. Festival) leaves and fruits were compared by high-performance liquid chromatography coupled with a diode array detector (UHPLC-DAD) and mass spectrometry (HPLC-MS). The total polyphenol strawberry leaf extracts exhibited a 122-fold-higher total polyphenol content and 13-fold higher antioxidant activity (ORAC) than strawberry fruits, and they showed evidence of possible photoprotective effects against UV damage in human melanoma cells (SK-MEL-28) and in murine embryo fibroblasts (NIH/3T3), together with promising anti-proliferative activities against the same melanoma cells. Seven polyphenols were confirmed by HPLC-DAD in the leaf extracts, with differences depending on fraction solubility. Moreover, three substituted quercetin derivatives, three substituted kaempferol derivatives, two anthocyanins, and catechin were confirmed in the soluble fraction by HPLC-MS. Given their higher total polyphenol content and bioactive activities, underutilized strawberry Festival leaves are a potential source of apparently abundant biomass with prospective bioactive applications.

## 1. Introduction

Commercial strawberries (*Fragaria x ananassa*) are one of the world’s most available and consumed fruits. By 2020, the global production of strawberries reached over 12 million tons in 79 countries [1], with an average annual consumption (as of 2018) of 4.5–5 kg per capita in the United States and Europe, respectively [2]. As an extensive crop, it also generates presumably large amounts of residues. Non-marketable fruit (small-sized and misshapen berries, with pest and disease damage or albinism) may reach up to 17% of total yields [3], and the vegetative parts of the plant (leaves, stems, and roots) are mostly discarded [4]. Moreover, around 21% of the strawberry fruit production is transformed for manufacturing processed products, yielding additional industrial organic wastes (mostly pulp and press residues) [5], all of which still contain relevant amounts of bioactive compounds [5,6,7,8].

All strawberry plant components (*Fragaria* sp.) have been shown to provide several benefits for human health (reviewed extensively in [9,10,11,12,13,14,15,16]); however, research has focused mainly on the fruit, while the biochemical profile and the biological activities of the vegetative parts have been less studied [17,18,19,20]. Plenty of these healthy effects have been associated with the polyphenol content in strawberry fruit; nevertheless, recent studies have shown more diversity and higher concentrations of bioactive polyphenols in strawberry leaves (Table 1) [17,18,20,21,22].

Berry leaves have been used in traditional remedies claiming to treat several diseases, including colds, gastroenteritis, diabetes, and circulatory ailments; however, berry leaf medicinal products have only recently been formally accepted in Europe, the United States, and Canada, and it mostly refers to the use of berry leaves in herbal teas [23,24]. To the best of our knowledge, no human studies with strawberry leaf interventions have been reported to date; nevertheless, the European Medicines Agency [25] communicated in 2019 that, based on long-term traditional practice, strawberry leaves can be used to induce urination and to relieve symptoms of mild diarrhea.

In vitro experiments with strawberry leaf extracts have reported several biological activities, including cytotoxic effects against leukemia HL60 human cells [26]; antioxidant activity [27,28]; antibacterial activity [29]; anti-allergic and anti-obesity effects [22]; and melanogenesis inhibition [22]. The animal testing of strawberry leaf extracts has shown hypoglycemic effects in diabetic mice [30] and rats [19]; antioxidant, anti-inflammatory, and cognitive-improvement effects in diabetic rats [31]; nephrotoxicity protection in cisplastin-dosed rats [32]; and improved responses in renal function, insulin, and free thyroid hormones in potassium bromate-treated rats [33]. Duru et al. [34] also showed no adverse effects on the egg quality and health status of laying hens after strawberry leaf powder intake for 10 weeks. 

Hence, strawberry leaves are a broadly available source of valuable and underutilized bioactive materials that can be harvested yearly after fruiting [24]. In this study, we explored the phenolic profile and bioactivity of *F. x ananassa* cv. Festival fruit and leaf extracts. Strawberry Festival is a short-day vigorous cultivar, with an apparent lesser susceptibility to botrytis fruit rot (caused by *Botrytis cinerea*) and to powdery mildew (caused by *Sphaerotheca macularis*) compared to that of other varieties [35]. Strawberry Festival has also shown a higher total polyphenol content in leaves than other strawberry cultivars (Table 1). To the best of our knowledge, this is the first report describing the leaf polyphenols of strawberries cultivated in the Costa Rican tropical highlands.

**Table 1:** Total polyphenol content in the fruit and leaves of some strawberry (*Fragaria x ananassa*) varieties.

Cultivar	Total Polyphenols (mg GAE g^−1^ DW) ^a^		Ref.
	Fruit	Leaf	
Allstar	19.71 ± 0.20	55.2 ± 3.6	[28]
Amaou	15.6 ± 3.7	117.1 ± 2.7	[22]
Camino Real	1.8 ± 0.11 (FW)	6.14 ^b^	[4,36]
Diana	-	14.76	[18]
Elsanta	24.11	3.73 ^c^	[37]
Festival	20 ± 0.1	62.37 ± 0.1	[19]
Mount Everest	-	293.63 ± 1.95	[27]
Polka	2.57 ± 0.02 (FW)	81.15 ± 0.64 ^c^	[21,38]
Red Merlin	33.33 ± 0.1	72.1 ± 0.1	[19]
San Andreas	-	8.93 ^b^	[4]
Senga Sengana	15.2 (FW)	326.57 ± 0.82	[27,39]
Suzana	35.24 ± 0.3	72.63 ± 0.1	[19]
Tamar	21.1 ± 0.5	42.63 ± 0.2	[19]
Tochiotome	-	122.3	[40]
Winter Dawn	31.42 ± 0.1	66.58 ± 0.2	[19]

^a^. DW, dry weight; FW, fresh weight; GAE, gallic acid equivalents. ^b^. Industrial strawberry by-products, including sepals, peduncles, and the non-marketable portion of the fruit. ^c^. Total sum of several polyphenols quantified by analytical methods as mg g^−1^ DW.

## 2. Results

### 2.1. Preliminary Phytochemical Analysis of Total Polyphenol Fruit and Leaf Extracts

To analyze the phytochemical components of strawberry (*F. x ananassa* cv. Festival) leaves, three hydro-alcoholic extraction methods were preliminary tested, i.e., maceration at room temperature with 70% or 95% ethanol and infusion at 60 °C with 70% ethanol, yielding 3.85 ± 0.08 g (*n* = 3), 1.7 g, and 3.23 g recovery of the total dried leaf extract (from a 10 g sample), respectively. The 70% ethanol maceration had the highest total polyphenol recovery relative to the 95% ethanol maceration and the 70% ethanol infusion (104.1 and 49.18 mg GAE g^−1^ DW, respectively). On the other hand, the 95% ethanol maceration had slightly higher total flavonoid recovery (12.99 mg QE g^−1^ DW) relative to the 10.25 mg GAE g^−1^ DW obtained with both the 70% ethanol maceration and the 70% ethanol infusion. Nonetheless, the 70% ethanol maceration showed the highest antioxidant activity, requiring less of the extract (DPPH IC_50_ 9.45 mg g^−1^ DW) to achieve 50% DPPH inhibition than the other extractions (DPPH IC_50_ required 14.32 mg g^−1^ DW of 95% ethanol leaf extract and 11.51 mg g^−1^ DW of 70% ethanol leaf infusion). Moreover, alkaloids were absent only in the 70% ethanol maceration leaf extract (data not shown). Hence, the 70% ethanol maceration was selected for extracting total polyphenols from strawberry leaves and for comparison against fruit.

The total polyphenol content in the total polyphenol strawberry (*F. x ananassa* cv. Festival) extracts (70% ethanol) was 122 times higher in leaves than in fruits, while the antioxidant activity was 13-fold higher in the leaves (Table 2). Only very small amounts of chlorophylls and carotenoids were recovered from both parts of the plant. Saponins were not detected in the fruit, while steroids and terpenoids were not detected in the leaf. Alkaloids were not detected in either of the plant extracts.

### 2.2. Chromatographic Analysis

**Figure 1:**
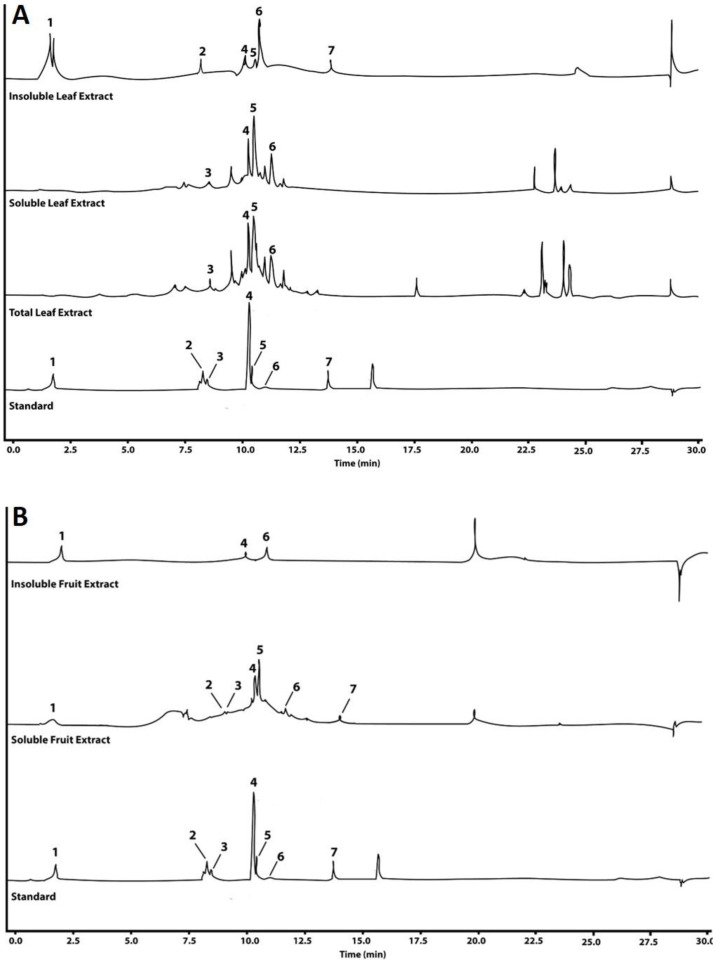
Representative UHPLC-DAD chromatograms of the polyphenols identified in the total, soluble, and insoluble polyphenol extracts of the strawberry (*Fragaria x ananassa* cv. Festival) leaf (**A**) and fruit (**B**) compared to authentic standards. The identity of the polyphenols is as follows: 1, gallic acid; 2, caffeic acid; 3, chlorogenic acid; 4, *p*-coumaric acid; 5, rutin; 6, ellagic acid; 7, quercetin.

Seven polyphenols were quantified by UHPLC-DAD in total, soluble, and insoluble polyphenol extracts from strawberry (*F. x ananassa* cv. Festival) fruit and leaves (Figure 1, Table 3). The highest polyphenol concentrations were found in the insoluble leaf extract, with gallic acid being the most abundant polyphenol, followed by ellagic acid and quercetin. On the contrary, gallic acid and quercetin were not detected in the soluble leaf fraction. When detected, all compounds were present in higher concentrations in the soluble and insoluble leaf extracts relative to the fruit. All seven polyphenols were present in the total polyphenol leaf extract, but in much lower concentrations than in the soluble and insoluble leaf fractions.

**Figure 2:**
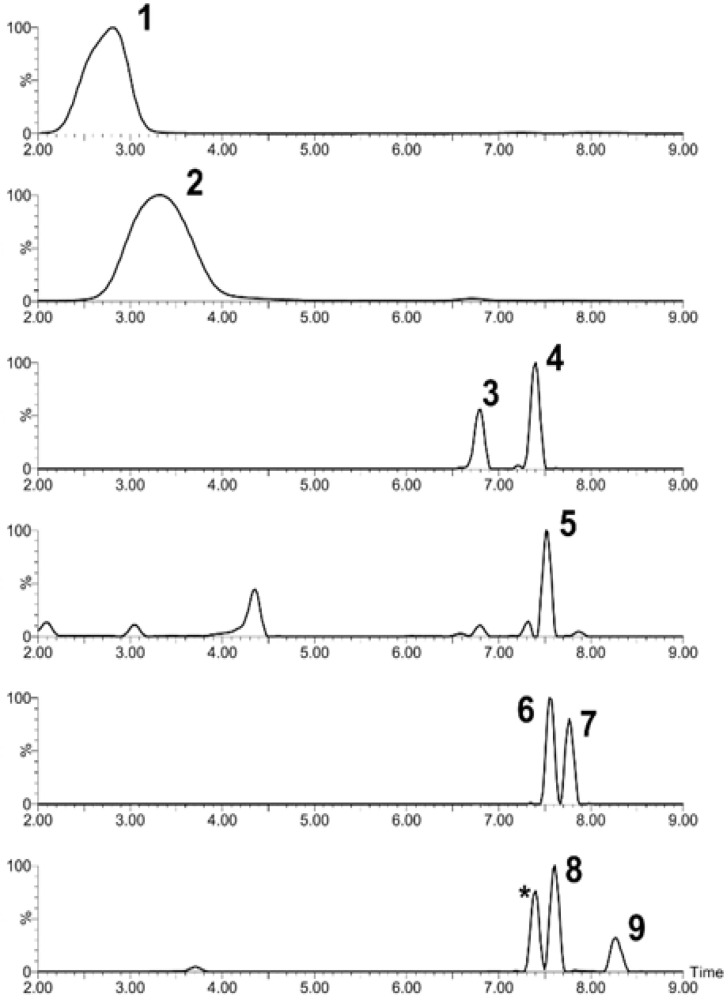
Combined extracted ion chromatograms of the most intense signals present in the high-resolution mass spectrometry measurements of the strawberry (*Fragaria x ananassa* cv. Festival) soluble leaf extract. Peak identification is detailed in Table 4. 1, catechin; 2, pelargonidin; 3, 4, and 8, quercetin glucuronides; 5, delphinidin; 6, 7, and 9, kaempferol derivatives. The asterisk corresponds to the aglycone in-source fragment of compound 4.

Untargeted high-resolution mass spectrometry was used to identify the polyphenols present in the soluble strawberry leaf extracts. A tentative identification was assigned to the more intense features present in the samples by comparison with the structure database searches and de novo fragmentation patterns and confirmed by manual interpretation. Nine compounds were identified, including three substituted quercetin derivatives, three substituted kaempferol derivatives, two anthocyanins (delphinidin and pelargonidin), and catechin (Figure 2, Table 4).

### 2.3. Cytotoxic Activity

**Figure 3:**
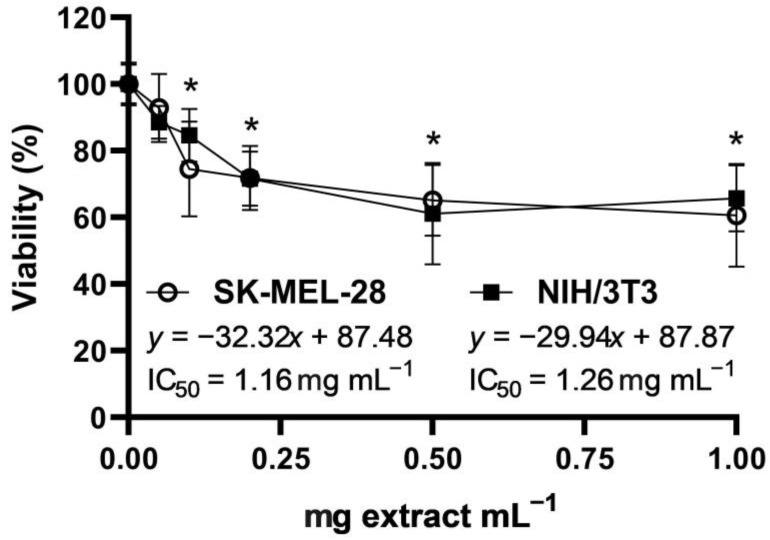
Percentage of cell viability (neutral red assay, mean ± SD, *n* = 9) of SK-MEL-28 (human melanoma) and NIH/3T3 (murine embryo fibroblasts) cells after 24 h of treatment with total polyphenol strawberry (*Fragaria x ananassa,* cv. Festival) leaf extract (0.05, 0.1, 0.2, 0.5, and 1 mg extract mL^−1^). Concentrations (in both cell lines) statistically different (*p* < 0.05) from the untreated control are marked by asterisks (one-way ANOVA followed by a Bonferroni’s post-test, GraphPad Prism, v. 9.4.1). IC_50_, half-maximal inhibitory concentration.

The cytotoxic effect (neutral red assay) of the total polyphenol strawberry leaf extract was evaluated in SK-MEL-28 human melanoma cells and in NIH/3T3 murine fibroblasts. Extract concentrations above 0.1 mg extract mL^−1^ significantly reduced the cell viability relative to the untreated controls in both cell lines (Figure 3). The extract’s half-maximal inhibitory concentrations (IC_50_) were very similar in both cell lines. 

### 2.4. UVA + UVB Photoprotection

**Figure 4:**
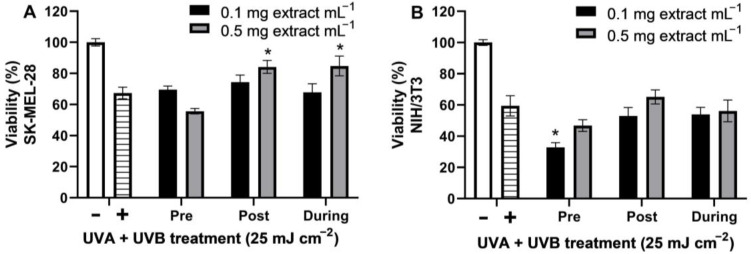
Percentage of cell viability (MTT assay; mean ± SEM, *n* = 6) of SK-MEL-28 (human melanoma, **A**) and NIH/3T3 (murine embryo fibroblasts, **B**) cells after treatment with total polyphenol strawberry (*Fragaria x ananassa*, cv. Festival) leaf extract (0.1 and 0.5 mg extract mL^−1^) before (24 h), after (24 h), or during (15 min) UV irradiation (UVA + UVB 25 mJ cm^−2^). Controls of untreated cells (white bars) and UV-only-treated cells (striped bars) are also shown. Extract treatments that significantly (*p* < 0.05) affected cell viability relative to the UV controls are marked with an asterisk (one-way ANOVA followed by a Bonferroni’s post-test, GraphPad Prism, v. 9.4.1).

SK-MEL-28 melanoma cells and NIH/3T3 murine fibroblasts were treated with the total polyphenol strawberry leaf extract before, after, or during UV irradiation (UVA + UVB 25 mJ cm^−2^). The UV dose reduced the cell viability to 67.40 ± 2.53% and 59.49 ± 3.75% in SK-MEL-28 and NIH/3T3 cells, respectively (Figure 4). The total polyphenol strawberry leaf extract (0.5 mg extract mL^−1^) significantly increased the cell viability of UV-irradiated SK-MEL-28 cells treated with the extract after or during UV irradiation. In the NIH/3T3 fibroblasts, pre-treatment with the total polyphenol strawberry leaf extract (0.1 mg extract mL^−1^) significantly decreased the cell viability of the UV-irradiated cells.

### 2.5. Scratch Wound Healing Assay

**Figure 5:**
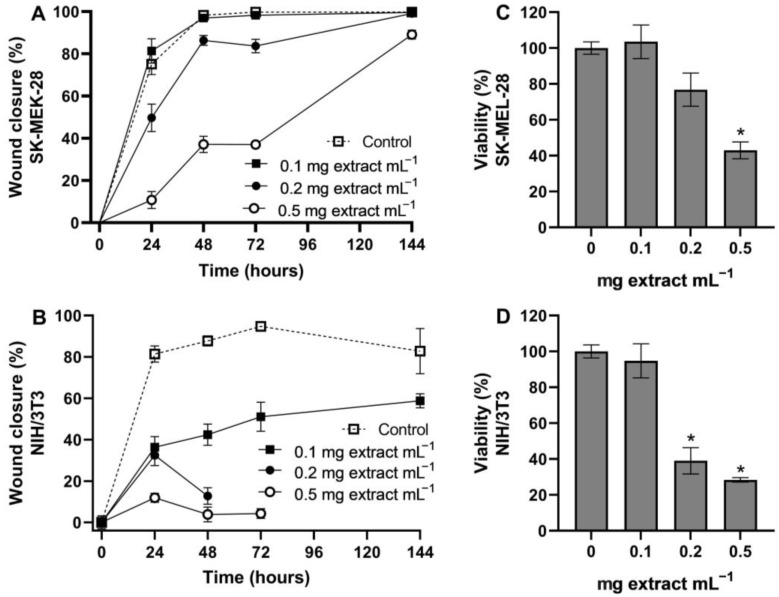
Scratch Wound Healing assay on scraped SK-MEL-28 (human melanoma (**A**,**C**)) and NIH/3T3 (murine embryo fibroblasts (**B**,**D**)) monolayers treated with total polyphenol strawberry (*Fragaria x ananassa,* cv. Festival) leaf extract (0.1, 0.2, 0.5 mg extract mL^−1^) for 72 h. Afterwards, the extract was removed, and the cells were kept in fresh media for another 72 h to allow for recovery. (**A**,**B**). Percentage (mean ± SEM, *n* = 6) of cell confluence in the lesion over time, calculated as the area covered with cells using the software Image J. (**C**,**D**). Percentage (mean ± SEM; *n* = 9 for SK-MEL-28, *n* = 6 for NIH/3T3) of cell viability (MTT assay) 72 h after replacing the extract with fresh media. Statistical differences (*p* < 0.05) in cell viability between the treatments and the untreated control are marked with an asterisk (one-way ANOVA followed by a Bonferroni’s post-test, GraphPad Prism, v. 9.4.1).

Wound closure was evaluated on scraped SK-MEL-28 and NIH/3T3 monolayers treated with the total polyphenol strawberry leaf extract for 72 h. None of the extract treatments significantly improved the cell migration or proliferation in either of the cell lines. In fact, the murine fibroblast monolayers fully detached after 48 h with the higher concentrations and did not recover after the leaf extract was removed (Figure 5). In the SK-MEL-28 melanoma cells, the leaf extract delayed the wound closure, but the lesion almost fully recovered after 72 h in extract-free media, except in the 0.5 mg extract mL^−1^ treatment. 

## 3. Materials and Methods

### 3.1. Extract Preparation

**Total polyphenol extraction.** Ripe strawberry fruits and adult strawberry leaves (*Fragaria x ananassa* cv. Festival) were purchased from Fresas de Altura S.A. in Llano Grande, Cartago, Costa Rica (permit R-CM-ITCR-001-2022-OT-CONAGEBIO). The samples came from a greenhouse where they were treated with ozone against plagues. The plant material was healthy, with no observable morphological or pathological alterations. The leaves were green in color and approximately 8 × 5 cm in size. Extraction was conducted as reported before [41]. Freeze-dried whole fruits or leaves were ground in a food processor (fruits) or in a mill (leaves; 1 mm, MF-10 basic IKA, WERKE) and stored at −20 °C until extraction. The dried material was extracted by three consecutive water-ethanol macerations (1:10, plant material to solvent). The solvent was eliminated in a vacuum evaporator (40 °C, R-300, BUCHI), and the remaining concentrate was freeze-dried and stored at −70 °C.

**Soluble and insoluble polyphenol extraction.** Following the procedure by Lux et al. [42], soluble free and conjugated polyphenols were extracted from 100 mg of the same freeze-dried plant material, mixed with 3 mL of aqueous methanol (80%, *v*/*v*) for 1 min in an ultrasonicator (Sonoplus, Bandelin, Germany), and centrifuged at 1718× *g* for 10 min. The supernatant was transferred to a fresh tube, and the extraction was repeated with 2 mL of aqueous methanol (80%, *v*/*v*) two more times. The combined supernatants were dried in a centrifugal vacuum-evaporator (Vacufuge, Eppendorf, Hamburg, Germany). For recovering insoluble bound polyphenols, the solid remainder from the soluble extraction was resuspended in 2 mL aqueous NaOH solution (2 mol L^−1^) and stirred for 4 h at 20 ± 1 °C. The solution was acidified (6 mol L^−1^ HCl) to pH 2, 2 mL of purified water was added, and three consecutive extractions were performed with ethyl acetate, following the same conditions mentioned above. 

### 3.2. Phytochemical Analysis

The total polyphenol fruit and leaf extracts were preliminary evaluated for the detection of flavonoids (Shinoda test), steroids and terpenoids (Liebermann–Buchard test), alkaloids (Dragendorff test), and sapponins (foam test) [43,44]. Soluble solids were determined in a refractometer (ATAGO) as the percentage °Brix.

The total polyphenol content (TPC) was determined by the Folin–Ciocalteu spectrophotometric method, as described by Rojas-Garbanzo et al. [45]. Fruit and leaf extracts were incubated with Folin–Ciocalteu reagent and sodium carbonate solution (75 g L^−1^) at 50 °C for 15 min, and the absorbance was measured at λ = 620 nm (FLUOstar OPTIMA, BMG LABTECH, Ortenberg, Germany). TPC was calculated against an external calibration curve of gallic acid (10–80 mg GAE L^−1^, *r*^2^ = 0.9909), and it is expressed as µg of gallic acid (>99% purity, Sigma-Aldrich, St Louis, MO, USA) equivalents (GAE) per gram of extract. The results are shown as the mean ± SD (*n* = 3). 

The total flavonoid content (TFC) was analyzed by the aluminum chloride method described by Fernandes et al. [46]. Fruit and leaf extracts (10 mg extract mL^−1^ in methanol) with 2% aluminum chloride solution (in methanol) were incubated for 10 min at room temperature. The absorbance was measured at λ = 450 nm (FLUOstar OPTIMA, BMG LABTECH, Ortenberg, Germany), and TFC was calculated against an external calibration curve of quercetin (0–160 mg QE L^−1^, *r^2^* = 0.9981) and is expressed as mg of quercetin (>99% purity, Sigma-Aldrich, St Louis, MO, USA) equivalents (QE) per gram of extract. The results are shown as the mean ± SD (*n* = 3).

To determine the chlorophyll and carotenoid content, the UV-VIS spectrum was recorded at λ = 200–750 nm (Shimadzu UV1800), with dimethyl-sulfoxide (DMSO) as a blank probe. The pigment content based on the absorbance was calculated as previously described [47], as follows:

$$Chlorophyll\;A\;\left(\mu g/\mathrm{mL}\right)=12.47\;A_{665.1}-3.62\;A_{649.1}\;Chlorophyll\;B\;\left(\mu g/\mathrm{mL}\right)=25.06\;A_{649}-16.5\;A_{665.1}\;Total\;carotenoids\;\left(\mu g/\mathrm{mL}\right)=\frac{1000\;A_{480}-1.29\;C_{A}-53.78\;C_{B}}{220}$$

The protein content was analyzed using the Quick Start^TM^ Bradford Protein Assay Kit (BIO-RAD), following the manufacturer’s protocol. The total protein concentration was calculated by comparing it against an external bovine serum albumin (BSA) standard curve (0–2 mg mL^−1^, *r*^2^ = 0.973).

The antioxidant activity was determined by the DPPH (2,2-diphenyl-1-picrylhydrazyl) radical scavenging assay, as described by Wang et al. [48]. Leaf samples (10 mg extract mL^−1^ in 70% ethanol) diluted in methanol were incubated with DPPH (0.5 mM in methanol) at 37 ºC for 30 min in the dark. Absorbance was measured at λ = 544 nm (FLUOstar OPTIMA, BMG LABTECH), and the radical scavenging activity is shown as the DPPH percentage inhibition = [Ac − As − Ab)]/(Ac × 100), where Ac is the absorbance of the negative control (DDPH without sample), As is the absorbance of the sample, and Ab is the absorbance of the sample without DPPH. The IC_50_ (the concentration of the sample required to inhibit the DPPH response by 50% with respect to the untreated control) was calculated from the linear equation of each curve (Graph-Pad Prism, v. 9.1.1; GraphPad Software, San Diego, CA, USA). The ORAC (Oxygen Radical Absorbance Capacity) was determined in the fruit and leaf total polyphenol extracts by the QUIMED Laboratory at Universidad Nacional de Costa Rica, based on the method described by Zamora et al. [49].

### 3.3. Chromatographic Analysis

For the polyphenol quantification, soluble and insoluble strawberry fruit and leaf extracts were analyzed as previously reported [42,50], with slight modifications. The samples were dissolved in 1 mL methanol HPLC-grade, membrane-filtered (0.20 μm PTFE), and injected (5 μL) in an ultra-high-performance liquid chromatography system (UHPLC) coupled with a diode-array detector (DAD, Ultimate 3000, series TQH-E1-0288, Thermo Fisher Scientific, Waltham, MA, USA). Polyphenols were separated on an Acquity UPLC CSH C18 column (1.7 μm particle size, 100 × 2.1 mm, Waters) maintained at 30 °C, using acetic acid aqueous solution (7.5 mM, eluent A) and acetonitrile (eluent B) at a flow rate of 0.25 mL min^−1^. The elution gradient was as follows: isocratic 95% A for 0.8 min, from 95% to 80% A in 5.2 min; isocratic 80% A for 0.5 min, from 80 to 70% A in 1 min; isocratic 70% A for 0.6 min, from 70 to 50% A in 6.9 min, from 50 to 0% A in 3 min; isocratic 0% A for 7.5 min, going back to 95% A in 0.1 min; and running isocratic at 95% A until a 30 min total run. Peaks were recorded and integrated using Chromeleon software (version 7.3, Thermo Fisher, Waltham, MA, USA)and identified and quantified using UV-VIS wavelengths and external curves of authentic polyphenols standards (Sigma-Aldrich, St Louis, MO, USA). At least four replicates were carried out per sample, and each one was injected once for UHPLC-DAD analysis.

Strawberry leaf soluble polyphenol extracts were also analyzed to obtain their untargeted phytochemical profile using high-resolution mass spectrometry coupled with liquid chromatography. Reconstituted samples were measured using a Xevo G2-XS quadrupole time of flight mass (Q-tof) spectrometer (Waters Corporation, Wilmslow, UK) coupled with an Acquity UPLC H-Class. A 5 µL injection was separated with a BEH-C18 column (2.1 × 100 mm, 1 µm) using a mobile phase gradient of A (water, 0.05% formic acid) and B (acetonitrile, 0.05% formic acid), setting a flow of 0.5 mL min^−1^. The gradient consisted of maintaining 5% B for 5 min, increasing to 80% at 10 min and subsequently to 100% at 11 min, and holding until 13 min to finish equilibrating the column to the initial conditions. The column temperature was set to 50 °C. The mass spectrometer was configured to use a capillary voltage of 2 kV, a 40 V sampling cone, and a source offset of 80 V. The source temperatures were set at 125 °C and 600 °C for the desolvation temperature, and the gas flows were set to 150 L h^−1^ for the cone gas and to 1000 L h^−1^ for the desolvation gas. Measurements were made using the MSe acquisition mode in positive and negative polarities, with a mass range from 50 to 1000 *m/z*, a scan time of 0.25 s, and a ramp of collision energy from 20 V to 40 V for the high-energy function.

The data were analyzed using Progenesis QI v 2.4 software with the Progenesis MetaScope for compound identification, using the referenced databases for polyphenols in food (Phenol-Explorer) [51] and flavonoids (MetabolomicsJP, Arita Laboratory, Uchiyama, Japan) [52]. Compound identification was assigned by matches, considering a mass error lower than 5 ppm, an over 85% isotopic distribution similarity, and the assignment by de novo fragmentation analysis of at least four fragments with a mass error lower than 5. The resulting compounds from the Progenesis QI search were manually curated and evaluated against the MassBank available fragmentation spectra. Tentative identifications were assigned to the matched compounds and described by the compound subclass.

### 3.4. Cell Viability Assay

SK-MEL-28 (passages 59–70) human melanoma cells (ATCC HTB-72) and NIH/3T3 (passages 20–30) murine fibroblasts (ATCC CRL-1658) were cultured in DMEM (4.5 g/L glucose, Sigma-Aldrich) supplemented with 10% FBS (Sigma-Aldrich), 1% L-glutamine (4 mM; GIBCO), 1% sodium pyruvate (0.11 mg/mL; Sigma-Aldrich), and 1% antibiotics (10,000 IU/mL penicillin and 10,000 μg/mL streptomycin; GIBCO, Grand Island, NY, USA). All cells were maintained at 95% humidity and 5% CO_2_ at 37 °C.

The cytotoxic effect of the total polyphenol strawberry leaf extract in the SK-MEL-28 melanoma cells and the NIH/3T3 normal murine fibroblasts was measured by the neutral red uptake assay according to Repetto et al. [53]. The cells were seeded onto 96-well plates (3 × 10^4^ cells per well) and treated in triplicate for 24 h, with the total polyphenol strawberry leaf extract (0.05, 0.1, 0.2, 0.5, and 1 mg extract mL^−1^) diluted in a supplemented culture medium. Neutral red dye (40 μg mL^−1^, Invitrogen) incorporated into the lysosomes was measured at λ = 540 nm (FLUOstar OPTIMA, BMG LABTECH). A linear dispersion curve of the percentage of cell viability relative to the untreated control was calculated, from which the half-maximal inhibitory concentration (IC_50_) was determined. The data are shown as the mean ± SD (*n* = 9). Each concentration was compared against the untreated control by one-way ANOVA, followed by a Bonferroni’s post-test (GraphPad Prism, v. 9.4.1, GraphPad Software, San Diego, CA, USA).

### 3.5. Photoprotection Experiment

SK-MEL-28 melanoma cells and NIH/3T3 normal murine fibroblasts were seeded on 24-well plates (1.48 × 10^6^ cells per well) and treated with total polyphenol strawberry leaf extract (0.1 and 0.5 mg extract mL^−1^) before (24 h in culture medium), after (24 h in culture medium), or during UV irradiation (UVA + UVB 25 mJ cm^−2^ in HBSS). Cell viability was measured 24 h after UV irradiation using the MTT assay, as described before [54]. The MTT formazan salts absorbance was measured at λ = 570 nm (FLUOstar OPTIMA, BMG LABTECH), and the percentage of cell viability relative to an untreated control was calculated. The data are shown as the mean ± SEM (*n* = 6). The effect of each treatment was compared against a control of UV-irradiated cells by a one-way ANOVA, followed by a Bonferroni’s post-test (GraphPad Prism, v. 9.4.1, GraphPad Software, San Diego, CA, USA).

### 3.6. Scratch Wound Healing Assay

To evaluate the effect of the total polyphenol strawberry leaf extract on the cell migration and proliferation, SK-MEL-28 and NIH/3T3 cells were seeded in 24-well plates (1.48 × 10^6^ cells per well). A cross-shaped flat wound was scratched in confluent monolayers with a sterile 1000 μL plastic micropipette tip, and monolayers were treated for 72 h with total polyphenol strawberry leaf extract (0.1, 0.2, and 0.5 mg mL^−1^). The monolayers were photographed every 24 h, and the percentage wound area over time was calculated using Image J (https://imagej.nih.gov/ij/, accessed on 15 October 2022). The extract was then removed, and cell viability was measured after 72 h of incubation with fresh media by the MTT assay, as described before [54]. The data are shown as the mean ± SEM. The effect of each concentration on cell viability (MTT assay) was compared against the untreated control by a one-way ANOVA, followed by a Bonferroni’s post-test (GraphPad Prism, v. 9.4.1, GraphPad Software, San Diego, CA, USA).

## 4. Discussion

This study compared the phenolic profiles of *Fragaria x ananassa* cv. Festival fruit and leaves from crops cultivated in Costa Rican tropical highlands, showing a 122-fold-higher total polyphenol content and 13-fold higher antioxidant activity (ORAC) in leaves than in fruits, respectively (Table 2). The total polyphenol leaf extracts (70% and 95% ethanol maceration and 70% ethanol infusion) showed the recovery of flavonoids by all extraction protocols, while steroids and terpenoids were absent in all treatments. The 70% ethanol maceration resulted in the highest crude yield (33.76%), the best total polyphenol recovery, and the highest antioxidant activity, and it was the only method that resulted in alkaloid-free extracts. Similar yields (38%) were obtained by El-Hawary et al. [19] for strawberry leaves using a similar extraction method. Thus, the extract obtained from 70% ethanol maceration at room temperature was selected for the general phytochemical comparison against fruits (Table 2) and for cell culture experiments (Figure 3, Figure 4 and Figure 5).

Other authors [17,18,19,22] have also shown higher polyphenolic contents in strawberry leaves compared to those in fruits, mostly ellagitannins. To further characterize the polyphenol profile in strawberry (*F. x ananassa* cv. Festival) fruit and leaves, seven polyphenols (gallic acid, caffeic acid, chlorogenic acid, *p*-coumaric acid, rutin, ellagic acid, and quercetin) were evaluated by UHPLC-DAD (Figure 1, Table 3) in soluble and insoluble polyphenol extractions, also showing higher concentrations in the leaf extracts relative to the fruit. Notably, gallic acid and rutin were the most abundant polyphenols in the insoluble fruit and leaf extracts and in the soluble and total leaf extracts, respectively.

The specific profiles of bioactive polyphenols in strawberry fruit and leaves (Table 1) exhibit wide variations depending on the location, genetics, ripening, growing methods, and harvesting and storage conditions, and the results are dependent on the analytical methods used in each investigation. Nonetheless, anthocyanins, which are rarely found in strawberry leaves [18], have been consistently reported as the most abundant phenolic compounds in strawberry fruits [8,55,56,57,58], followed by flavanols, catechins, and procyanidins [55]. In contrast, ellagitannins are the most abundant polyphenols usually reported in strawberry leaves [20,21,24], representing ca. 47.0−54.3% of the total leaf phenolic content [21] and exhibiting up to 14-times-higher total ellagitannins in strawberry leaves (151.78 ± 10.66 mg g^−1^ DW) relative to the fruit (10.73 ± 1.49 mg g^−1^ DW) [20]. Agrimoniin has been reported as the most abundant ellagitannin in several strawberry varieties [18,20,21]. Other authors have reported cinnamic acid, ferulic acid, and quercetin exclusively in strawberry fruit (cv. Lucy, Joly, Darselect, Selvik, Diana, and Clery), while ellagic acid, kaempferol, and rutin were present only in the leaves of the same varieties [18]. In contrast, Skupień and Oszmiański [59] found quercetin only in the leaves of six strawberry varieties (cv. Kent, Elsanta, Senga, Selva, Elkat, and Dukat). The same authors also observed ellagic acid in the fruit, but in concentrations four times lower than those in the leaves, and the most abundant polyphenols in these varieties were not ellagitannins but *p*-coumaric acid [59]. Gallic acid, caffeic acid [26], and chlorogenic acids [21,24] have also been reported before in strawberry leaves. Several other phytochemicals have been reported to be present in strawberry leaves but absent in the fruits, including terpens and plant steroids [19], which have been less studied and warrant further investigation.

**Figure 6:**
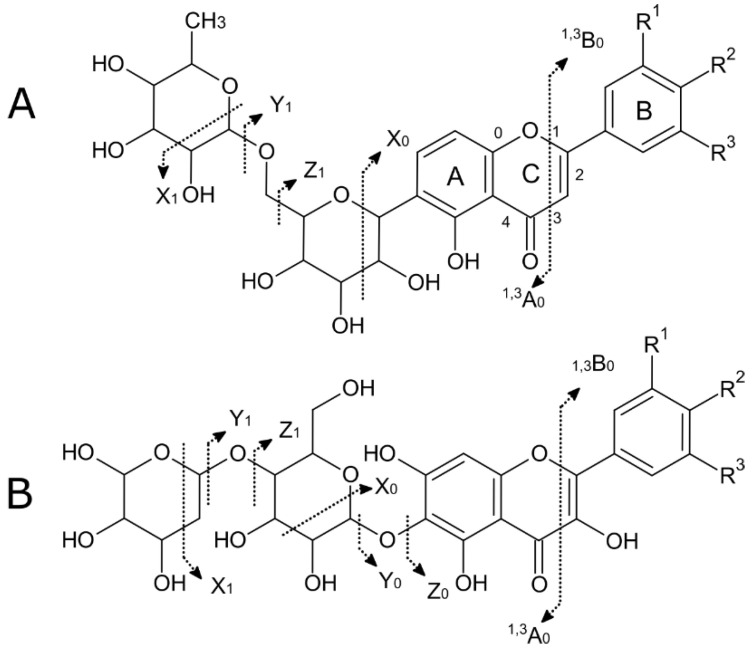
Nomenclature and common product ions of flavones and flavonols C-glycosides (**A**) and O-glycosides (**B**).

Some evident peaks remained unidentified in the soluble and total leaf extracts (Figure 1). Due to the lack of purified standards and the vast diversity of the structural isomers of flavonoids, the identification of these compounds in plant extracts is a challenge that needs strategies for the proper assignment of tentative identifications [60]. High-resolution mass spectrometry allows for the assignment of an elemental composition to adduct ions within 5 ppm of the mass error, and, combining it with tandem fragmentation spectra, it is possible to assign a putative identification to relevant features. Following the nomenclature by Domon and Costello [61] for proposed flavonoid glycosides, it is possible to characterize fragmentation patterns to obtain more specific information about flavonoids aglycones substituents (Figure 6). This allowed for the identification of different flavonoids families in the soluble leaf extract: flavonols, flavans, flavones, and anthocyanins.

Due to the cleavage of O–C bonds, glycosylated flavonoids present a fragmentation leading to Y_0_^−^ or Y_0_^+^ ions as the main product ions. In negative and positive ionization modes, the formation of ions losses for an O-hexose [M-162 ± H]^+/−^, O-deoxyhexose [M-146 ± H]^+/−^, and O-pentose [M-132 ± H]^+/−^ allows for the determination of the glycan size and structure [60]. Additionally, the characteristic product ions of acyl groups commonly occurring in the glycosyl part of flavonoids are also key to the assignment of a tentative structure to detected features. Neutral losses for malonyl [M+H-86]^+^, galloyl [M+H-152]^+^, gallic acid [M+H-170]^+^, coumaroyl [M+H-146]^+^, and feruloyl [M+H-176]^+^, among others, complement the assignation of defined identifications [62].

**Figure 7:**
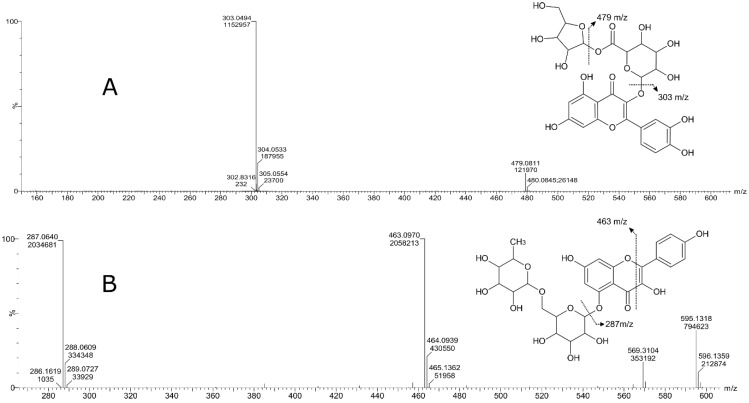
Fragmentation spectra of selected compounds ((**A**) compound 4; (**B**) compound 6) with the assignment of putative identifications and fragmentation patterns.

Accordingly, compound 2 (Figure 2, Table 4) presents a 271 *m*/*z* ion as a neutral loss of 162 corresponding to the loss of a hexose from the glycosylated pelargonidin. Compound 3 presents fragmentation ions 465 *m*/*z* and 303 *m*/*z* corresponding to feruloyl [M+H-176]^+^ and O-hexose [M-162+H]^+^ neutral losses, where the 303 *m*/*z* ion corresponds to the protonated quercetin aglycone. Compound 4 was also assigned to a quercetin aglycone that presented 479 *m*/*z* and 303 *m*/*z* fragments corresponding to O-pentose [M-132+H]^+^ and feruloyl [M+H-176]^+^ neutral losses, respectively (Figure 7A). Compound 5 presents a neutral loss corresponding to the loss of one O-hexose [M-162+H]+ from an anthocyanin glycoside. Compound 6 presented the formation of A_0_ and Y_0_ fragments, kaempferol 7-O-rutinoside being the only known structure to present that possible elemental composition considering the formation of the 287 *m*/*z* product ion of the protonated kaempferol aglycone (Figure 7B). Compound 7 presented an [M+H-176]^+^ neutral loss due to the presence of a glucuronide, and the designation of a kaempferol aglycone was confirmed by pseudo MS^n^ spectra with the fragmentation of the 287 *m*/*z* ion and its comparison to the MassBank reference MS/MS spectra of lutein and kaempferol, as kaempferol presents fragments with masses of 165 *m*/*z* and 153 *m*/*z*, as observed for compound 7. Compound 8 was identified as a quercetin 3-glucuronide with an [M+H-176]^+^ neutral loss, and compound 9 was identified as a kaempferol 3-(6′-p-coumaroyl glucoside) with the neutral loss of the coumaryl galactoside group [M+H-162-146]^+^.

Previous studies have suggested the potential use of strawberries in cosmetic and pharmaceutical topical products, providing possible antioxidant, photoprotective, and anti-aging activities [63,64,65,66,67]. Hence, the bioactivity of the total polyphenol leaf extract was evaluated in terms of cytotoxicity, UV-photoprotection, and wound healing potential in human melanoma cells (SK-MEL-28) and in murine embryo fibroblasts (NIH/3T3). The extract exhibited half-maximal inhibitory concentrations (Figure 3) within the range considered as inactive (IC_50_ > 1000 μg mL^−1^) [68], suggesting low cytotoxicity in these cell lines. Nonetheless, the cell viability at concentrations above 0.1 mg leaf extract mL^−1^ was already significantly lower than that of the untreated control in cell lines that are highly proliferative. Therefore, experiments with normal human epidermal and dermal cells are required to better evaluate the concentration limits of the strawberry leaf extract when considering future topical applications.

Polyphenols may be successful photoprotectors since their structure is comparable to that of organic UV filters, thus protecting cell viability by absorbing UV radiation [69]. They may act as a sunscreen, lessening the oxidative stress, inflammation, and DNA damage caused by UV radiation in the skin [70]. Notably, post-treatment with plant polyphenols has prevented the UV-induced upregulation of inflammatory and metabolic responses in NHEK normal human keratinocytes more effectively than pre-treatment, affecting mainly delayed cellular events as opposed to primary photochemical reactions [71]. Our total polyphenol strawberry leaf extract aided the recovery of melanoma cells treated with the extract after or during UV irradiation and reduced the survival of the murine fibroblasts pre-treated with the extract (Figure 4), suggesting a possible increase in repair and defense mechanisms, a dampening of the UV damage to the cells, and an inhibition of cells with genotoxic damage, respectively. Future investigations should explore molecular markers to further evaluate how the leaf extract modulates oxidative stress, DNA damage, DNA repair, and cell death in UV-damaged normal human cells.

The total polyphenol strawberry leaf extract also delayed or inhibited wound healing in both cell lines (Figure 5). Although in conflict with the possibility of using this extract in topical formulations, these results also suggest a potential for strawberry leaves to inhibit or delay cancer cell proliferation and migration, which could be useful as a co-adjuvant in anti-cancer treatments. Additionally, the anti-proliferative effect was stronger in the normal murine fibroblasts than it was in the human melanocytes; thus, full tissue permeability must be considered in future studies to determine if topical applications may be restricted the epidermis.

Interestingly, Zhu et al. [22] reported total polyphenols in strawberry roots (179.2 ± 13.0 mg GAE g^−1^ DW) and flowers (181.8 ± 13.3 mg GAE g^−1^ DW) in similar or even higher concentrations than those in the leaves (117.1 ± 2.7 mg GAE g^−1^ DW), as well as ca. three-times-higher antioxidant activity in the flowers. This suggests that other underutilized parts of the strawberry plant may also hold relevant bioactive and commercial potential, which is of interest for future studies.

## 5. Conclusions

Given their higher total polyphenol content and superior antioxidant activity relative to fruits, underutilized strawberry (*Fragaria x ananassa*) cv. Festival leaves from crops cultivated in Costa Rican tropical highlands are a potential source of apparently abundant biomass with relevant bioactive potential, including possible photoprotective and anti-proliferative activities. However, before considering eventual topical applications, future experiments with normal human epidermal and dermal cells and tissues are necessary to evaluate aspects such as the absorption, distribution, metabolism, and stability of the leaf components, as well as in understanding the molecular mechanisms involved in normal skin tissue responses to this extract. 

## Figures and Tables

**Table 2:** General physicochemical composition of strawberry (*Fragaria x ananassa* cv. Festival) fruit or leaves in total polyphenol extracts (*n* = 3).

Parameter	Fruit Extract	Leaf Extract
Protein (mg g^−1^ DW)	28.63 ± 2.09	80.63 ± 1.13
Soluble solids (° Brix)	1%	0.40%
Saponins (foam test)	Negative	Positive
Steroids and terpenoids (Liebermann–Buchard test)	Positive	Negative (no color)
Alkaloids (Dragendorf test)	Negative (no color)	Negative (no color)
Flavonoids (Shinoda test)	Positive (light red)	Positive (red color)
Total polyphenols (mg GAE g^−1^ DW)	0.89 ± 0.05	108.83 ± 4.65
Total flavonoids (mg QE g^−1^ DW)	0.32 ± 0.01	10.25 ± 0.50
Total chlorophyll (mg g^−1^ DM)	0.2832 ± 0.0224	1.9470 ± 0.6399
Total carotenoids (mg g^−1^ DW)	0.0013 ± 0.0020	0.0074 ± 0.0933
ORAC (µmol TE g^−1^ DW)	221 ± 70	2918 ± 281

GAE, gallic acid equivalents; QE, quercetin equivalents; TE, trollox equivalents. ORAC was determined by the QUIMED Laboratory at Universidad Nacional de Costa Rica.

**Table 3:** Specific polyphenols (mean ± standard deviation; *n* = 4) detected and quantified by UHPLC-DAD in the total, soluble, and insoluble polyphenol extracts of the strawberry (*Fragaria x ananassa* cv. Festival) fruit and leaves.

Peak	Compound	Retention Time (min)	UV/Vis Wavelength (nm)	Polyphenol Concentration in [µg mg^−1^ DW] in the Extract				
				Insoluble Extract		Soluble Extract		Total Extract
				Fruit	Leaf	Fruit	Leaf	Leaf
1	Gallic acid	2.06	201/220/270	2.10 ± 1.40	57.53 ± 36.84	0.04 ± 0.01	n.d.	0.0010 ± 0.0001
2	Caffeic acid	8.51	216/240/322	n.d.	3.62 ± 4.97	0.14 ± 0.12	n.d.	0.0028 ± 0.0002
3	Chlorogenic acid	8.69	215/320	n.d.	n.d.	0.17 ± 0.18	0.66 ± 0.31	0.20 ± 0.02
4	*p*-coumaric acid	10.51	209/310	1.50 ± 0.56	3.70 ± 2.68	0.21 ± 0.06	1.08 ± 0.09	1.14 ± 0.23
5	Rutin	10.63	201/256/355	n.d.	7.14 ± 3.40	0.58 ± 0.07	4.67 ± 0.70	5.59 ± 2.69
6	Ellagic acid	11.15	196/254/366	0.11 ± 0.21	21.90 ± 39.72	0.26 ± 0.09	1.35 ± 0.08	0.88 ± 0.57
7	Quercetin	13.91	201/255/370	n.d.	19.44 ± 3.98	0.05 ± 0.09	n.d.	0.098 ± 0.003

n.d. = not detected.

**Table 4:** Tentative identification of the most intense features present in the high-resolution mass spectrometry measurements of the strawberry (*Fragaria x ananassa* cv. Festival) soluble leaf extract.

Peak	Compound Subclass	Tentative Identification	Formula	*m*/*z*	Adducts	Rt (min)	Assigned Fragments (*m*/*z*)
**1**	Flavan 3-ol derivatives	Catechin	C_15_H_14_O_6_	291.0887	[M+H]+	2.91	273, 165, 147,139, 123
**2**	Pelargonidin 3-glucoside	Pelargonidin 3-glucoside	C_21_H_21_O_10_^+^	433.1130	M+	3.32	433, 271
**3**	Flavonoid-3-*o*-glucuronides	Quercetin 3-glucosyl-(1->2)-glucuronide	C_27_H_28_O_18_	641.1355	M+H, M+Na	6.8	641, 465, 303
**4**	Flavonoid-3-*o*-glucuronides	Quercetin 3-*o*-xylosyl-glucuronide	C_26_H_26_O_17_	611.1324	[M+H]+	7.4	611, 479, 303
**5**	Anthocyanin glycoside	Delphinidin 3-galactoside	C_21_H_21_O_12_^+^	465.1051	M+	7.51	465, 303
**6**	Flavonoid *o*-rutinoside	Kaempferol 7-*o*-rutinoside	C_30_H_26_O_13_	595.1318	M+H	7.55	595, 463, 287, 153, 113
**7**	Flavone *o*-glucoside	Kaempferol 3′-glucuronide	C_21_H_18_O_12_	463.0876	M+H, M+Na	7.76	436, 287, 165, 153
**8**	Flavonoid-3-*o*-glucuronides	Quercetin 3-glucuronide	C_21_H_18_O_13_	479.0827	M+H, M+Na	7.61	303, 301, 179, 151
**9**	Flavonoid 3-*o*-p-coumaroyl glycoside	Kaempferol 3-(6′′-*p*-coumarylgalactoside)	C_30_H_26_O_13_	595.1461	M+H	8.24	287, 147, 119

## Data Availability

The datasets from the current study are available from the corresponding author on reasonable request.

## Sample Availability

Not available.
